# Convergence between parent report and direct assessment of language and attention in culturally and linguistically diverse children

**DOI:** 10.1371/journal.pone.0180598

**Published:** 2017-07-06

**Authors:** Kerry Danahy Ebert

**Affiliations:** Department of Communication Disorders & Sciences, College of Health Sciences, Rush University, Chicago, IL, United States of America; Birkbeck College, UNITED KINGDOM

## Abstract

Parent report is commonly used to assess language and attention in children for research and clinical purposes. It is therefore important to understand the convergent validity of parent-report tools in comparison to direct assessments of language and attention. In particular, cultural and linguistic background may influence this convergence. In this study a group of six- to eight-year old children (*N* = 110) completed direct assessments of language and attention and their parents reported on the same areas. Convergence between assessment types was explored using correlations. Possible influences of ethnicity (Hispanic or non-Hispanic) and of parent report language (English or Spanish) were explored using hierarchical linear regression. Correlations between parent report and direct child assessments were significant for both language and attention, suggesting convergence between assessment types. Ethnicity and parent report language did not moderate the relationships between direct child assessments and parent report tools for either attention or language.

## Introduction

Parent report is a crucial component in assessment for many developmental disabilities. It can provide historical and functional perspectives not otherwise available in a clinical assessment setting. Collecting parent report is also consistent with the fundamental healthcare shift towards patient-reported measures [1]. However, parent report tools should demonstrate convergent validity with direct clinical assessments of children, meaning that the two types of measures should be related for the assessment of any given developmental disorder. It is critical to understand not only how each specific tool corresponds with direct clinical assessments but also the factors that influence the convergent validity of parent reports in general. In particular, the ever-increasing diversity of clients in many clinical settings necessitates an understanding of the possible influence of culture and language on parent report tools.

This study explored the convergence between parent report and direct clinical assessment of the symptoms of two common developmental disorders: attention deficit-hyperactivity disorder (ADHD) and language learning impairment (LLI; also known as language disorder, specific language impairment, primary language impairment, and language-based learning disability; see [2] for discussion). Although both disorders are high-incidence and share a high co-morbidity rate [3], the role of parent report in the assessment process for the two disorders is sharply different. In the present study, a group of 110 school-age children with diverse racial, ethnic, and linguistic backgrounds completed language and attention assessments and a parent completed an interview for each child, which included questionnaires regarding both language and attention. Within this dataset, we explore the relations between parent report and child performance, with a focus on the influence of cultural-linguistic diversity on these relations.

### Developmental disorders of attention and language

ADHD and LLI are two of the most common developmental disorders, although they differ significantly in terms of how they are diagnosed and managed. ADHD is characterized by persistent symptoms of inattention, hyperactivity-impulsivity, or both; common symptoms include failure to follow through on tasks, losing important items, becoming distracted easily, moving excessively, and acting impulsively [4]. It is typically diagnosed using ratings of behavioral symptoms collected from parents, teachers, or affected individuals, although complete diagnostic criteria specify that these symptoms must be present across multiple settings [4]. Direct assessments of children’s attention skills, such as continuous performance tests, exist as a potential complement to symptom checklists [5]. Recent national survey data indicate that 8.4% of parents report that their child has received a diagnosis of ADHD at some point [6]. However, meta-analysis of research on prevalence has indicated the true rate may be lower (around 5.9–7.1%) [7].

In contrast, LLI is characterized by difficulty developing language skills at the expected rate. Common symptoms include grammatical errors in speaking or writing, reduced vocabulary size and depth of vocabulary knowledge, and difficulty comprehending spoken or written discourse such as stories. The language difficulties in LLI are not explained by intellectual delay, sensory impairment, or another neurological condition (such as autism spectrum disorder). LLI is typically identified through direct assessments of children’s language skills, such as standardized tests and language samples [8, 9]; symptom checklists and other parent report instruments exist as a potential complement to direct language assessment [10]. In children who speak more than one language, LLI will manifest as slow development in all languages, and thus it is critical to assess all languages spoken by bi- or multi-lingual children with suspected LLI [11]. The prevalence of LLI, based on direct assessment of language, is approximately 7.4% in kindergarten children [12].

Although the common diagnostic processes for these disorders differ, direct clinical assessments and parent report tools exist for both disorders. Information on the correspondence between these tools is limited, particularly for school-age children. Because the tools purport to measure the same construct (either attention or language), they should correspond across the range of children’s abilities (i.e., within both average and impaired performance bands). The next section reviews existing literature on the correspondence between parent report and direct child assessment for both ADHD and LLI, with a focus on the tools of interest in the present study.

### Convergent validity of parent report on attention

As noted above, the use of parent-reported symptomatology is widespread in the diagnostic process for ADHD. Although multiple scales exist, the focus of the current study is on a specific commonly used instrument, the Vanderbilt ADHD Diagnostic Parent Rating Scale (VADPRS) [13]. The VADPRS was developed to directly correspond to the 18 core symptoms of ADHD that are specified in the DSM-IV [14]. The scale has strong internal consistency and corresponds well to a more in-depth diagnostic interview for ADHD [14, 15]. However, convergent validity with direct child assessments (such as continuous performance tests) has not been explored for the VADPRS.

Outside of the VADPRS specifically, however, there is a body of literature considering the correspondence between parent-reported information and direct child assessment of attention skills, particularly using continuous performance tests. For example, Epstein and colleagues [5] considered the relations between ADHD symptoms, as measured by a structured parent interview corresponding to the DSM-IV criteria for ADHD, and child performance on a continuous performance test in a large epidemiological sample of 9- to 17-year old children. Several outcome measures from the test (including signal detection measures and variability in reaction time) were significantly related to almost all 18 parent-reported ADHD symptoms. In contrast, Edwards et al. [16] found no significant relationships between the same tools in a smaller sample. Other work has supported the convergence between various forms of parent-related symptoms and continuous performance test measures [17, 18, 19], though discussion remains over whether these two types of tools measure identical constructs.

There is a very limited literature examining the convergence between parent-reported ADHD symptoms and the specific continuous performance task used in this study, the Test of Variables of Attention (TOVA) [20]. In the validation study for the original version of the TOVA [21], no significant correlations were reported between TOVA measures and the Attention Problems score from the Child Behavior Checklist, a parent-report instrument. More recently, Wu and colleagues [19] studied a relatively small group (N = 61) of Taiwanese children with and without diagnosed ADHD. They reported a significant correlation between parent-reported hyperactivity symptoms, again as measured by the Child Behavior Checklist, and one overall outcome measure from the TOVA. In short, the literature on convergence between parent report and direct child assessments (particularly continuous performance tests) is conflicting and limited, particularly for the tools of interest in this study (i.e., the TOVA and the VADPRS).

### Convergent validity of parent report on language

There is a robust literature suggesting that parents of toddlers can accurately rate their children’s level of language development; parent estimates of early language skills such as vocabulary correspond highly with direct child assessment of these same skills [22, 23]. Moreover, these parent estimates of language skill are predictive of language development and accurate in identifying the presence of early language delay (typically, a precursor to LLI) [24, 25].

These investigations have largely been limited to children aged one or two years, however. For children beyond this age range, parent report instruments for language assessment exist but have less empirical support. As children develop, their language skills grow exponentially (thus becoming more difficult to directly observe) and they typically spend smaller proportions of time with their parents (reducing parents’ ability to directly observe). It is possible, then, that parent report and direct child assessment of language diverge as children age.

Although the majority of studies comparing parent-reported language skill, particularly for the purpose of clinical assessment, have considered toddlers, there also exists a significant literature considering parent report in preschool and school-age children who speak a minority home language. This literature has focused on two major questions. First, studies have addressed whether parent report can be used to establish the amount of input a child receives in both the home language (typically called the first language or L1) and the community language (typically the second language or L2) [26]. Parent reports of proportional input and output generally correspond with direct child assessment of proficiency in the languages spoken [26, 27].

The second focus of this literature has been on the use of parent report to assess development and identify LLI in a minority home language. Although LLI has traditionally been identified on the basis of test performance, there are pragmatic reasons to rely more heavily on parent report in this population. First, the diversity of language experiences among minority L1/majority L2 learners makes this group incomparable to the normative sample of most diagnostic language tests [28], invalidating the tests for the purpose of identification of LLI. Secondly, there is a profound shortage of service providers who can directly assess minority languages, both within the United States and worldwide [29]. Thus, in many cases, the only means of obtaining information about the development of a minority L1 is via parent report.

The limited evidence to date suggests that parent report contributes to accurate identification of LLI in minority L1 learners. Restrepo [30] investigated measures that could discriminate LLI from typical language development in a group of 62 predominantly Spanish-speaking children, aged 5 to 7 years, in the United States. Measures included a set of 29 parent questions regarding their children’s current speech or language problems and 21 additional parent questions on family history of speech-language problems; both of these parent-reported measures demonstrated strong discriminant accuracy. In particular, both measures showed very high specificity (at 95.7% for current speech-language problems and 91.3% for family history). More recently, Paradis, Emmerzael, & Sorenson Duncan [31] sought to validate a new parent questionnaire, the Alberta Language Development Questionnaire (ALDeQ) [31]. The ALDeQ contains sections on early developmental milestones, current abilities in the L1, behavioral patterns, and family history of speech, language or learning difficulties. Paradis et al. tested its ability to discriminate between children with and without LLI in a group of 168 five- to eight-year old children from a range of L1 backgrounds. As with Restrepo’s results [30], the parent questionnaire was a significant discriminator between children with and without LLI, but it showed stronger specificity than sensitivity. The section on early developmental milestones showed the strongest relationship with current language status.

It is important to note that both of these studies sought to discriminate between previously identified children with LLI and their typically-developing peers, rather than to consider the correspondence between parent report and direct child assessment across a range of language abilities. The literature on parent reports in school-age children would benefit from additional consideration of this correspondence, particularly across diverse groups of children.

### Cultural and linguistic influences on parent report in diverse populations

The work described above speaks to the general convergent validity of parent reports for children with ADHD or LLI. However, it is important to acknowledge the potential role of cultural and linguistic differences when tools are applied across diverse groups of parents. Although the literature on minority home language learners suggests that parent report on language is useful for identifying LLI and for capturing language exposure patterns, other sources acknowledge the potential for cultural or linguistic influences on these reports [23, 32]. Tools designed for use with one population of parents (most often, speakers of the majority language who identify with the majority culture) may not maintain their properties when completed by parents outside this population.

One challenge in collecting parent report across diverse populations is linguistic; that is, parents may not fluently speak (or read) in the language of the tool. Clearly, questions may be misinterpreted or inaccurately answered in this scenario. One common solution is to translate the parent report tools. For example, multiple Spanish translations of the VADPRS are readily available [e.g., 33]. Although translating parent report tools increases their utility in linguistically diverse populations, it also alters the tools’ properties—potentially introducing bias [32]. One particular difficulty for language development questionnaires is translating language-specific milestones, such as the use of specific grammatical forms.

A second challenge in collecting parent report across diverse populations is cultural. A family’s degree of acculturation within the culture reflected on the parent report tool may influence responses [23]. Cultural expectations may be apparent in the questions, such as when questions assume a specific style of parent-child interaction [32]; for example, in cultures with a high value on children’s obedience, questions about a child’s question-asking behavior may present a conflict for parents who highly value obedience. When parents are asked to judge whether behavior is problematic, as on many ADHD rating scales, cultural expectations may shape responses [33, 34].

There has been limited consideration of cultural and linguistic effects on parent report tools in both the ADHD and LLI literatures. For ADHD, a large-scale (n = 1478), pan-European study of the ADHD Rating Scale–IV [35] supported the factor structure of the tool across several different countries. However, significant differences in scores across countries–accounting for 15% of the variance in scores—were demonstrated. Cross-country differences in ADHD ratings have been reported in other studies as well [34]. There is also evidence that ADHD ratings by parents may differ across racial groups, with African American parents more likely to endorse hyperactive symptoms in their children [33]. Hillemeier and colleagues [33] attribute these differences to cultural influences as well as a possible lack of information about ADHD among minority parents.

For LLI, although the ALDeQ was designed and validated for parents from a range of linguistic backgrounds, Paradis et al. [31] did report potential differences in scores among the four major linguistic groups represented in the study. The Arabic group reported the lowest (i.e. poorest) scores, whereas the Cantonese/Mandarin group reported the highest. Without direct child assessment of these languages, it was not possible to further explore the nature of these differences. Additional investigations of differences in parent report across cultural or linguistic groups, particularly for school-age children, are limited and further study of this area is warranted.

### The current study

The purpose of this study was to explore the correspondence between parent-report tools and direct child assessments for both ADHD and LLI within a group of diverse school-age children. The following research questions are addressed:

1. a. Do parent-reported symptoms of ADHD converge with direct child assessment of attention skills?
  b. Does the convergence differ across parent groups defined by culture or by linguistic background?
2. a. Do parent-reported language skills and developmental history converge with direct child assessment of language skills?
  b. Does the convergence differ across parent groups defined by culture or by linguistic background?

## Materials and methods

This study was approved by the Rush University Medical Center Institutional Review Board. Written consent to participate was obtained from the parents or guardians of all participants.

### Participants

A total of 110 children participated in the current study. The children were recruited for participation in a larger project examining attention skills in monolingual and bilingual children with and without LLI. Recruitment took place primarily at community-based after-school programs in neighborhoods with a high density of Hispanic families. Additional targeted recruitment of children with LLI occurred via school-based referrals.

There were 50 females and 60 males. All children were aged 6;0 to 8;11 at the time of participation; the mean age of participants was 7 years, 5 months. Children were exposed to either English only or both English and Spanish. Children with systematic exposure to other languages were excluded. Forty-nine children were classified as monolingual English speakers and the remaining 61 spoke both English and Spanish. Children who could converse and complete expressive and receptive language testing in both English and Spanish were considered bilingual. The rate of parental Spanish input, reported using the Alberta Language Environment Questionnaire [36] and averaged across the parents present in the home, ranged from 100% Spanish to 12.5% Spanish. Nearly all monolingual children (n = 42) came from homes in which there was no reported Spanish use. In the remaining 7 cases, parents reported a small percentage of parental Spanish input in the home (up to 12.5%), but the children were unable to produce any Spanish responses or complete basic instructions in Spanish when testing was attempted.

The participant sample was diverse in terms of social and economic circumstances. Reported maternal education levels ranged from “did not complete high school” (n = 33) to completion of a postgraduate degree (n = 11). Over half of the mothers in the sample (n = 65) reported an educational level of high school completion or lower. Eighty-six families reported their race as white, with an additional 20 reporting their race as African American and four families choosing not to report race. Seventy-nine families reported Hispanic ethnicity, 30 reported non-Hispanic ethnicity, and one family did not report this information.

Per parent report, children did not have prior histories of traumatic brain injury or seizures, and had no diagnoses of cognitive or intellectual disabilities, autism spectrum disorder, or cerebral palsy. Although the inclusion criteria for the larger study excluded children with formal diagnoses of ADHD, some parents expressed concern with attention skills when interviewed using the VADPRS. According to the VADPRS ratings, three children met criteria for the Predominantly Inattentive subtype of ADHD, three children met criteria for the Predominantly Hyperactive/Impulsive subtype, and one child met criteria for the combined subtype. Children’s language skills ranged from above average to impaired. Because the purpose of the larger study was to investigate the effects of LLI, children with LLI were deliberately recruited from school and clinical settings. A total of 30 children met criteria for LLI by demonstrating depressed scores on language testing (in both Spanish and English for children with bilingual exposure) along with the presence of parent or school concern about language skills.

### Measures

Two types of measures were employed in the present study: parent report measures and direct assessments of children’s skills.

#### Parent report measures

Parents completed three instruments during a telephone phone or in-person interview: the VADPRS, the ALDeQ, and the Alberta Language Environment Questionnaire [36]. The Alberta Language Environment Questionnaire was used to capture the proportion of Spanish used in the home environment. It was used to classify children as monolingual English-only or bilingual Spanish-English speakers, but is not explored further in the current study.

The VADPRS asks parents to rate the frequency of 18 behaviors that correspond to the symptoms of ADHD. Nine items represent symptoms of inattention and nine items represent symptoms of hyperactivity and impulsivity. Parents also rate children’s performance in school, family and peer relationships, and participation in organized activities. Additional VADPRS items corresponding to symptoms of Oppositional-Defiant Disorder, Conduct Disorder, and Anxiety/Depression were not administered in this study. Scores for Inattention and Hyperactivity can be calculated by summing ratings across the nine items in each domain, and a total symptom score is obtained by summing ratings across both domains. The University of North Carolina translation of the VADPRS [37] was used for Spanish-speaking parents.

The ALDeQ [31] asks parents 19 questions across four sections: early developmental milestones, current language abilities, activity patterns and preferences, and family history of communication and learning difficulty. Questions in the current language abilities section are written to refer to the “language of the home country”, as the tool was originally designed for use across a range of immigrant populations. For the current study, questions in that section were modified to refer specifically to either Spanish or English (according to the language predominantly used in the home). The ALDeQ generates proportion scores for each of the four sections, as well as an overall score. Fluent study staff translated the ALDeQ into Spanish to use with Spanish-speaking parents.

#### Direct child assessments

Children completed three measures to assess language and attention skills. To assess English language skills, all children completed the Clinical Evaluation of Language Fundamentals– 4th Edition (ECELF) [38]. The ECELF is considered an omnibus measure of language ability. For children aged 6–8 years, four subtests make up a core language score: Concepts & Following Directions, in which children listen to instructions of increasing length and complexity and respond via pointing; Word Structure, in which children complete sentences eliciting target grammatical structures in a cloze task format; Recalling Sentences, in which children repeat sentences of increasing length and complexity; and Formulated Sentences, in which children compose sentences using a target word. Each subtest yields a scaled score and the four subtests can be combined to yield a standard Core Language score. The normative sample of the ECELF is composed of monolingual English-speaking children in the United States.

Children with systematic exposure to Spanish completed the Spanish version of the Clinical Evaluation of Language Fundamentals– 4^th^ Edition (SCELF) [39]. For 6 to 8 year-old children, the test contains Spanish correlates of the English subtests (Conceptos y Siguiendo Direcciones; Estructura de Palabras; Recordando Oraciones; Formulación de Oraciones). As with the ECELF, scaled subtest scores and overall Core Language standard scores can be obtained. The normative sample of the SCELF is composed of children in the United States who learned Spanish as a first language, with subsequent English exposure.

To assess sustained attention, children completed the visual TOVA (TOVA, 2013). The visual TOVA is a 22-minute continuous performance test using nonverbal stimuli. Children watch a computer screen for the appearance of boxes and are asked to respond to targets based on their spatial location on the screen. The overall index score from the TOVA, known as the Attention Comparison Score (ACS), combines response time, accuracy, and variability. The score is derived via comparison to a normative database, which includes children in the United States without behavioral concerns or special education services.

### Procedures

Children were tested in 60–90 minute sessions in a quiet space at their school, after-school program site, or the investigator’s laboratory. The TOVA, ECELF, and SCELF were conducted following the published protocols. The TOVA was administered on a laptop computer with a 12.5 inch screen.

Bilingual Spanish-English children (N = 61) completed the four Core Language subtests of both the ECELF and SCELF. Testing in English and Spanish was conducted on separate days, with the order of languages counterbalanced across participants. All language testing was conducted by trained examiners who were fluent in the language of test administration. Monolingual English-speaking children (N = 49) completed only the ECELF. For a small subset of these monolingual children (N = 9), there were no concerns with language development and only two subtests (Concepts & Following Directions and Recalling Sentences) were completed in order to verify the absence of language concerns. This was consistent with the larger study protocol, which allowed for quickly screening both receptive and expressive language skills in monolingual children with no identified concerns. The Recalling Sentences subtest was used because sentence repetition tasks are one of the single best diagnostic indicators of children with LLI [40]. The Concepts & Following Directions was used because it is the only one of the Core Language subtests that assesses receptive language. For the remaining monolingual English speaking children, all four of the Core Language subtests were administered.

To create a single language test score for analyses, *z* scores were created separately for monolingual and bilingual children. For bilingual children, assessment of both languages is recommended, but a single language score was needed for analyses. Therefore core language scores in English and Spanish were averaged to obtain one overall score encompassing both languages. This was transformed to a *z* score using the mean and standard deviation of the bilingual sample. For monolingual children, the Core Language score in English was used. For the subset of children who completed only 2 subtests, a Core Language score was created by averaging the scaled scores from the two subtests and then extrapolating to the standard (vs. the scaled) score distribution based on the published norms. For example, for a child with scaled scores of 7 and 8, the average scaled score of 7.5 would fall 0.83 standard deviations below the mean on the scaled score distribution (in which 10 is the mean and 3 is the standard deviation); on the standard score distribution (in which 100 is the mean and 15 is the standard deviation), the corresponding score is 87.5. Scores from monolingual children were then transformed into *z* scores using the mean and standard deviation of the monolingual sample.

Parent interviews were conducted via phone or in-person, with the interviewer recording responses on paper scoring sheets for the VADPRS and ALDeQ. Parents were offered the option to complete the interview in Spanish or in English; 51 parents chose a Spanish interview and 61 chose an English interview. Study staff conducting the interviews were fluent in the language of the interview.

### Analyses

Before conducting the primary analyses, correlations between chronological age and the language and attention variables of interest were examined. This step was conducted because of the possibility that relations between parent report and direct child assessment could differ within the age range of the children in the study. Because some variables correlated significantly with age, chronological age was controlled for in all further analyses.

To explore the overall convergence of parent report with direct child assessment (Questions 1a and 2a), partial correlation analyses controlling for the effect of chronological age were performed. For attention, the parent report variables included the VADPRS scores for Inattention, Hyperactivity, and Total Symptoms. The direct child assessment variable was the ACS, or overall Attention Comparison Score, from the TOVA. For language, the parent report variables included the proportion scores for each of the four subsections of the ALDeQ (developmental milestones, current language abilities, activity patterns, and family history) and the total proportion score. The direct child assessment variable was the language testing *z* score.

To explore possible differences in convergence across cultural and linguistic groups (Questions 1b and 2b), hierarchical linear regression analyses were performed. Each analysis endeavored to predict a direct child assessment variable (attention or language) using one parent report variable, one cultural or linguistic variable, and the interaction between the parent report variable and the cultural or linguistic variable. The cultural variable used for these analyses was participants’ identified ethnicity (Hispanic vs. non-Hispanic) and the linguistic variable was the language of the parent interview (English vs. Spanish). The combination of two dependent variables of interest and two cultural or linguistic variables resulted in four regression analyses: attention as predicted by parent report, culture, and their interaction; attention as predicted by parent report, interview language, and their interaction; child language skill as predicted by parent report, culture, and their interaction; child language skill as influenced by parent report, interview language, and their interaction. For each analysis, chronological age, the parent report variable, and the cultural or linguistic variable were entered in the first model. The interaction term was then entered to examine the possible moderating effect of the cultural or linguistic variable on the convergence between the parent report variable and the direct child assessment variable.

## Results

Table 1 shows descriptive information on the attention and language measures used in the current study. Scores on the VADPRS inattention and hyperactivity scales spanned nearly the entire range of the instrument (i.e., from 0 reported symptoms up to a maximum possible symptom score of 27 on either scale). Similarly, proportion scores on each section of the ALDeQ spanned the entire possible range of the instrument (from 0 to 1).

**Table 1:** Descriptive statistics for parent and child measures.

Domain	Measure	Index	Mean	SD	Range
Attention	VADPRS	Inattention	6.28	4.91	0–24
		Hyperactivity	6.08	5.15	0–25
		Total	12.36	8.93	1.0–43.0
	TOVA	ACS	-1.12	3.17	-10.71–4.95
Language	ALDeQ	Milestones	0.80	0.29	0–1
		Current abilities	0.69	0.26	0–1
		Activities	0.73	0.14	0–1
		Family history	0.39	0.41	0–1
		Total	0.70	0.18	0.21–0.98
	Language	CELF *z* composite	0	1	-2.21–2.15

VADPRS scores for inattention and hyperactivity represent the sum of parent ratings from 0–3 across 9 symptoms. Total scores are the sum of the inattention and hyperactivity scores. TOVA ACS scores represent the composite attention score in comparison to the normative scores for the instrument; negative scores represent more difficulty with attention. ALDeQ scores are reported as proportions for each section and for the total parent report instrument. CELF scores are reported as *z* scores in comparison to the appropriate sample (monolingual or bilingual) in the current study. **Abbreviations**. VADPRS = Vanderbilt ADHD Diagnostic Parent Rating Scale; TOVA = Test of Variables of Attention; ACS = Attention Comparison Score; ALDeQ = Alberta Language Development Questionnaire; CELF = Clinical Evaluation of Language Fundamentals.

### Overall convergence between parent report and direct child assessment

The correlations among age, parent report variables from the VADPRS, and direct assessment of child attention skills via the TOVA are shown in Table 2. The bivariate correlations indicated that age was significantly related to the overall TOVA score, the VADPRS Inattention total, and the VADPRS total symptom score. Therefore, the convergence between parent report and direct child assessment was considered after controlling for age, in the partial correlations.

**Table 2:** Bivariate and partial correlations (age removed) among parent report and direct child attention measures.

		__Direct__	__________Parent Report (VADPRS)___		
		TOVA	Inattention	Hyperactivity	Total
	Age	-.47***	.28**	.09	.20*
Direct	TOVA		-.43***	-.18	-.36**
Parent Report (VADPRS)	Inattention	.36***		.58***	.88***
	Hyperactivity	-.15	.54***		.90***
	VAPRS Total	-.29**	.86***	.89***	

Bivariate correlations are displayed above the shaded diagonal, and partial correlations controlling for age are displayed below the shaded diagonal. TOVA score is the ACS or Attention Comparison Score. See Table 1 legend for abbreviations.

*** *p* < .001

** *p* < .01

* *p* < .05

After controlling for age, the direct assessment of child attention (i.e., the overall TOVA score) correlated significantly with the total symptom score of the VADPRS, *r (107)* = -.29, *p* < .01. However, there was a discrepancy in the relations between the TOVA and the two subscales of the VADPRS: the TOVA was significantly correlated with the VADPRS Inattention total, *r (107)* = -.36, *p* < .001, but not with the VADPRS Hyperactivity total, *r (107)* = -.15, *p* = .12. Because of this result, the VADPRS Inattention total was used as the parent report variable in subsequent regression analyses.

Correlations among age, parent report variables, and direct assessment of child language skills are shown in Table 3. Bivariate correlations with age were significant for one subscale of the ALDeQ parent questionnaire, the Milestones section, and for the ALDeQ total score. Partial correlations, correcting for the effects of age, were therefore considered in order to examine convergence between parent report on the ALDeQ and direct assessment of language using the composite *z* score from the CELF.

After controlling for age, the composite *z* score from language testing correlated significantly with all subsections of the ALDeQ. The strongest correlation occurred between the total ALDeQ score and the language composite *z* score, *r (107)* = .55, *p* < .001. Because the total ALDeQ provided the strongest correlation, it was used as the parent report variable in subsequent regression analyses.

**Table 3:** Bivariate and partial correlations (age removed) among parent report and direct child language measures.

		___Direct__	_________Parent Report (ALDeQ)__________				
		CELF Composite *z*	Milestones	Current abilities	Activities	Family history	Total
	Age	-.05	-.30**	-.14	-.18	-.02	-.24*
Direct	CELF Composite *z*		.34***	.45***	.42***	.27**	.55***
Parent Report (ALDeQ)	Milestones	.34***		.38***	.29**	.20*	.74***
	Current Abilities	.45***	.36***		.44***	.29**	.78***
	Activities	.42***	.25*	.43***		.23*	.62***
	Family History	.26***	.21*	.29**	.23*		.53***
	Total	.55***	.72***	.78***	.61***	.54***	

Bivariate correlations are displayed above the shaded diagonal, and partial correlations controlling for age are displayed below the shaded diagonal. See Table 1 legend for abbreviations.

*** *p* < .001

** *p* < .01

* *p* < .05

### Differences in Convergence Across Cultural And Linguistic Groups

#### Attention

To test possible differences in convergence across ethnic groups between parent report and direct child assessment of attention, a hierarchical linear regression to predict the overall TOVA score was conducted. In the first block of variables, age, the VADPRS Inattention total and participant ethnicity (Hispanic or non-Hispanic) were entered. This model was significant, *F* (3, 104) = 17.56, *p* < .001, and accounted for 34% of the variance in TOVA scores. Age contributed significantly to the model, *t(106)* = -5.11, *p* < .001. The VADPRS Inattention score also contributed significantly to the model, *t(106)* = -2.77, *p* = .007, but ethnicity marginally did not, *t(106)* = -1.98, *p* = .05. In the second block the interaction between VADPRS Inattention and ethnicity was entered in order to test the possible moderating effect of ethnicity. The resulting model remained significant, *F* (4, 103) = 13.06, *p* < .001, but the addition of the interaction term did not significantly improve the model, *∆R*^*2*^< .001, *p* = .83. Regression coefficients and *R*^*2*^change statistics appear in Table 4 for both models.

**Table 4:** Hierarchical linear regressions to predict child attention performance.

	____________Ethnicity____________						____________Language of Interview____________						
Variable	_____Model 1_____			_____Model 2_____			Variable	_____Model 1_____			_____Model 2_____		
	B	SE(B)	β	B	SE(B)	β		B	SE(B)	β	B	SE(B)	β
Age	-1.59	.31	-.43	-1.59	.31	-.43	Age	-1.44	.32	-.38	-1.46	.32	-.39
VADPRS Inattention	-0.16	.06	-.24	-0.17	.08	-.26	VADPRS Inattention	-0.22	.06	-.33	-0.18	.12	-.27
Ethnicity	1.13	.57	.16	0.98	.92	.14	Interview language	-0.39	.52	-.06	-0.09	.96	-.02
Interaction				0.02	.11	.03	Interaction				-0.05	.13	-.08
*R*^*2*^ change				.00			*R*^*2*^ change				.00		
Total *R*^*2*^	.34			.34			Total *R*^*2*^	.32			.32		

For each model, unstandardized coefficients appear in the B column, standard errors of the unstandardized coefficients appear in the SE(B) column, and standardized beta coefficients appear in the β column.

To test possible differences in convergence based on interview language, a second hierarchical linear regression to predict the direct assessment of attention (i.e., the overall TOVA score or ACS) was conducted. The first model included age, the VADPRS Inattention score, and the parent interview language (Spanish vs. English) as predictor variables. The resulting model was significant, *F* (3, 106) = 16.61, *p* < .001, and accounted for 32% of the variance in TOVA scores. Both age (*t(107)* = -4.54, *p* < .001) and the VADPRS Inattention score (*t(107)* = -3.91, *p* < .001) again contributed significantly to this model but parent interview language did not *t(107)* = -.75, *p* = .45. The interaction between VADPRS Inattention and parent interview language was entered into the second model. The resulting model remained significant, *F* (4, 105) = 12.39, *p* < .001, but the addition of the interaction term did not significantly improve the model, *∆R*^*2*^ = .001, *p* = .71. The regression coefficients and *R*^*2*^change statistics for these models also appear in Table 4.

#### Language

To test possible differences in convergence between parent report and child assessment of language skills across ethnic groups, a hierarchical linear regression was conducted using the overall language *z* score derived from the CELF as the dependent variable. In the first block, age, the total ALDeQ score and the participant ethnic group were entered into the model. The resulting model was significant, *F* (3, 105) = 12.74, *p* < .001, and accounted for 27% of the variance in language scores. The parent report score from the ALDeQ was significant in the model, *t(106)* = 6.10, *p* < .001, but age (*t(106)* = 0.74, *p* = .46) and ethnicity *t(106)* = 0.71, *p* = .48) were not. The interaction term between ALDeQ score and ethnicity was entered into the next model. This model remained significant, *F* (4, 104) = 9.46, *p* < .001, but the addition of the interaction term once again did not significantly contribute to the model, *∆R*^*2*^< .001, *p* = 1.0. Statistics for the regression models predicting language scores appear in Table 5.

**Table 5:** Hierarchical linear regressions to predict child language performance.

	_____________Ethnicity_____________						_____________Language of Interview_____________						
Variable	_____Model 1_____			_____Model 2_____			Variable	_____Model 1_____			_____Model 2_____		
	B	SE(B)	Β	B	SE(B)	β		B	SE(B)	β	B	SE(B)	β
Age	0.07	.10	.06	0.07	.10	.06	Age	0.08	.10	.07	0.08	.10	.07
ALDeQ Total	2.95	.48	.53	2.95	.75	.53	ALDeQ Total	3.14	.48	.56	3.31	.77	.59
Ethnicity	0.13	.18	.06	0.13	.70	.06	Interview language	-0.17	.16	-.09	0.02	.69	.01
Interaction				0.00	.96	.00	Interaction				-0.29	.97	-.11
*R*^*2*^ change				.00			*R*^*2*^ change				.00		
Total*R*^*2*^	.27			.27			Total *R*^*2*^	.29			.29		

For each model, unstandardized coefficients appear in the B column, standard errors of the unstandardized coefficients appear in the SE(B) column, and standardized beta coefficients appear in the β column.

Finally, to examine possible differences in convergence between parent report and children’s language scores based on the parent interview language, a fourth hierarchical linear regression was conducted. The dependent variable was the overall language *z* score, and the first model included age, the total ALDeQ score and the parent interview language as predictors. This model was significant, *F* (3, 106) = 14.71, *p* < .001, and accounted for almost 30% of the variance in language scores. Again, the parent report variable (total ALDeQ score) contributed significantly to the model, *t(107)* = 6.60, *p* < .001, whereas age and the linguistic diversity variable (interview language) did not (age: *t(107)* = 0.82, *p* = .41; interview language: *t(107)* = -1.06, *p* = .29). To test the possible moderating effect of interview language on the relationship between parent report and child assessment of language skills, the interaction term was added to the second model. As in the previous analyses, the model remained significant, *F* (4, 105) = 10.96, *p* < .001. The interaction term did not improve the model, *∆R*^*2*^ = .001, *p* = .77. The regression coefficients and the *R*^*2*^change statistics for these models appear in Table 5.

## Discussion

The overall goal of this study was to consider the convergent validity of parent report tools and direct child assessments in the areas of attention and language. Across both areas, the general convergence between these types of tools was positive and significant: with the exception of the VADPRS Hyperactivity scale, all subcomponents and overall scores on the parent report tools correlated significantly with the direct child assessments. Partial correlation coefficients, controlling for the effect of age, ranged from *r* = .26 to *r* = .55, representing medium to large effect sizes [41]. Thus, this study provides evidence that parent report and direct child assessment converge for both constructs of interest here, attention and language.

It should be noted, however, that even the highest correlation (*r* = .55 for the overall ALDeQ score with the language *z* score) indicates incomplete overlap between the parent report tools and the direct child assessments. This is perhaps unsurprising, given the very different means of collecting information about the construct of interest, but it is clear that the two tools do not index that construct in exactly the same manner. From a clinical assessment standpoint, the two scores might both contribute diagnostic information, but they are not interchangeable.

The role of age should also be considered. Age correlated significantly with the direct assessment of child attention (the TOVA), with one subscale and the total score on the parent report tool for attention (the VADPRS), and with one subscale and the total score on the parent report tool for language (the ALDeQ). For the parent report instruments, correlations were small to medium [41]; parents of younger children tended to report more symptoms of inattention and poorer early language development. The negative correlation between age and the overall TOVA score was larger and unexpected, because the TOVA ACS is normed for age.

It is important to note that the present study did not attempt to determine which type of instrument (parent report or direct child assessment) is more accurate in indexing the two constructs of interest. This represents a somewhat thorny issue, particularly given the differences in diagnostic procedures discussed earlier (i.e., reliance primarily on parent report instruments for ADHD vs. reliance on direct child assessment for language). If the parent report scores are viewed as the “gold standard” assessment for ADHD, then the results of this study suggest moderate convergent validity across a wide range of scores for the direct child assessment, the TOVA, with the caveat that age played an unexpected role in TOVA scores here. Conversely, if the direct child assessments are viewed as the gold standard assessment for LLI, the present results suggest moderate convergent validity across a wide range of scores for the parent report instrument, the ALDeQ. In both areas, however, these assumptions of gold standard assessments could easily be questioned. Thus, the present study refrains from judgments about the overall accuracy of the instruments, instead reporting only general convergence across a range of abilities.

### Differences in convergence across subcomponents

Within each construct, there were differences in the degree of convergence across the subcomponents of the parent report tools. In the area of attention, parent-reported inattention symptoms correlated significantly with direct attention assessment via the TOVA whereas parent-reported hyperactivity symptoms did not. This result conflicts with Wu et al. [19], who found significant correlations between parent-reported hyperactivity symptoms and the TOVA. One relevant methodological difference is that the present study did not attempt to recruit children with diagnosed ADHD (although some children who met diagnostic criteria did complete the study), whereas Wu et al [19] had groups of children with and without the disorder. It is not clear, however, why this difference would affect hyperactivity and impulsivity differentially, particularly considering that equal numbers of children in the present study met VADPRS criteria for the inattentive and hyperactive subtypes of ADHD. It is clear that additional work is needed to clarify the conflicting literature on parent report and direct assessment of attention using continuous performance tests such as the TOVA.

In the area of language, all subscales of the parent report measure, the ALDeQ, correlated significantly with the composite *z* scores from the direct child language testing. The largest correlation for a subscale occurred with the current abilities section, which asks parents to rate the child’s current communication skills in the home language. The result here indicates that parents are able to accurately rate their children’s language abilities, as measured by standardized language testing. The result is also consistent with the validation testing for the ALDeQ [31], which found that the current abilities section had the highest ability to discriminate between children with and without LLI. It echoes early recommendations to focus on ratings of the present rather than the past when developing parent-report assessments of language [22]. The family history section, in contrast, demonstrated the lowest correlation of any ALDeQ subscale with the composite *z* scores for language. This is again consistent with prior work on the ALDeQ [31].

### Differences in convergence across cultural and linguistic groups

The second set of research questions in this study explored the possible influence of cultural and linguistic variables on the convergence between parent report and direct child assessment. A series of hierarchical linear regressions were conducted to consider the moderating effects of reported ethnicity and of the parent’s preferred language for the interview on the relationship of interest for both language and attention. The results of these analyses were uniform: there was no evidence that either ethnicity or language influenced convergence in the assessment of either attention or language after controlling for age. This is an encouraging result, as it suggests that translating the parent report tools into Spanish did not alter their properties substantially. It also indicates that the tools performed similarly across two different ethnic groups, Hispanic and non-Hispanic.

It is important to inject a note of caution into these findings. First, the negative result here leaves open the possibility that convergence could differ between other ethnic or racial groups, for translations into languages other than Spanish, or for different assessment tools than the ones considered here. It is possible that the ethnic groups considered in this study were more similar to each other than were the varied immigrant groups in Paradis et al. [31] or the international sample in Döpfner et al. [35]; both of these studies found differences in scores across cultural groups. Race is another factor that may influence the performance of parent report tools (e.g., [33]). Because of the limited numbers of African American participants and because of the confound between ethnicity and race (i.e., all African American participants were also monolingual English speakers and non-Hispanic), the possible role of race was not explored here.

Further work is needed to illuminate the conditions under which culture, ethnicity, race, and language influence the performance of parent report tools. In the meantime, using culturally and linguistically appropriate assessment tools remains critical, and translations must be undertaken carefully to preserve the validity of the initial tool.

### Limitations

One potential limitation of the present study is that participants with LLI were intentionally recruited to the study sample whereas children with ADHD were not. This could potentially influence the range of scores on both parent and child tools, particularly for attention. There were, however, several children who scored in the impaired range on both the TOVA and the VADPRS, suggesting that the sample contained an adequate range of ability in the area of attention.

A second limitation is the unexpected relation between age and the overall TOVA score, which is derived from comparison to age-based norms. One possibility is that younger children in this sample had poorer attention skills in comparison to expectations for their age. Alternatively, it is possible that the normative data for the TOVA is imperfect for the youngest age groups included in the normative sample.

It was also necessary to transform the language testing scores to obtain a single language index from children who completed testing in two languages. Clinically, an average of scores across two languages should not be compared to a score obtained in a single language. However, the lack of significant differences in convergence across families that completed the interview in Spanish versus English suggests that the language *z* score calculations did not differentially affect convergence across the monolingual and bilingual groups.

### Conclusions

The present study explored convergence between parent report instruments and direct child assessments in the areas of attention and language. Significant and moderate convergence was found for almost all components of the parent report tools, and there was no evidence that cultural group or the translation of the parent report tools into Spanish influenced this convergence. These results are generally encouraging for the use of both types of tools in the assessment of attention or language for clinical or research purposes within diverse populations, although the unexpected role of age mitigated some convergence between the attention instruments. Additional work is needed to clarify the relationship between these types of tools, including their relative diagnostic accuracy and their performance across linguistic and cultural groups not studied here.
